# Dr. Wendell Holmes and the Secret of the Universe

**Published:** 1888-07-21

**Authors:** 


### DR. WENDELL HOLMES AND THE SECRET OF THE UNIVERSE.

SOME of our readers may not have read Dr. Oliver Wendell Holmes' account of how he one day discovered the secret of creation. Having observed that when unconsciousness is consciously approached, as during the inhalation of an anæsthetic—when the mind is on the confines of two worlds—there arise, sublime and voluminous, but fugacious thoughts; and having satisfied himself that in these thoughts, if they could ever be caught and transcribed, there lay enshrined the secret of the universe, he determined that by a supreme effort of the will he would catch and transcribe them. So, placing himself in his arm-chair, with pen, ink, and paper at hand, he inhaled the vapour of chloroform. As drowsiness stole over him, and just as unconsciousness was impending, those sublime and marvellous thoughts arose, and by a vigorous effort he seized his pen and wrote—he knew not what, for before he had finished he fell back unconscious. When he awoke, with trembling anxiety he turned to the sheet of paper, on which he could read, in characters rather scrawling, but quite legible, the secret of the universe expressed in these words: "A strong smell of turpentine pervades the whole."

